# 

**Published:** 1897-03-13

**Authors:** 


					THE HOSPITAL. ABSOLUTELY PURE, therefore BEST. March 13th, 1897.
CADBURY'S M fe* CADBURY'S
COCOA
" The standard of highest purity at present
attainable."?Lancet. NO ALKALIES USED
COCOA
^"UiAsaoir WesternIhfibmamx
JSTo.^S46j Vol. XXI. Lffw"] Saturday,
March 13th, 1897.
irr/bn fli/o/tUAi)
[SubsoriptionTerma,] pRICE TWOPENCE.

				

